# Use of Maribavir in Adult Patients With Post-Transplant Refractory Cytomegalovirus Infection in the Real-Life Setting

**DOI:** 10.3389/ti.2026.15769

**Published:** 2026-02-19

**Authors:** Nassim Kamar, Fanny Vuotto, Catherine Cordonnier, Philippe Gatault, Faouzi Saliba, Lionel Couzi, Vincent Bunel, Cinira Lefevre, Michèle Maric, Abdelkrim Ziad, Sophie Alain

**Affiliations:** 1 Department of Nephrology and Organ Transplantation, Axe TImE, Toulouse Rangueil University Hospital, INSERM UMR 1291, Toulouse Institute for Infectious and Inflammatory Diseases (Infinity), University Paul Sabatier, Toulouse, France; 2 Infectious Diseases Department, CHU Lille, Lille, France; 3 University Paris-Est-Créteil, Créteil, France; 4 Department of Nephrology-Hypertension, Dialysis and Renal Transplantation, Hôpital Bretonneau et Hôpital Clocheville, Tours, France; 5 Hepato-Biliary Centre, AP-HP Hôpital Paul Brousse, Villejuif, France; 6 University Paris Saclay, and INSERM No 1193, Gif-sur-Yvette, France; 7 Nephrology, Transplantation, Dialysis - Apheresis Department, Pellegrin Hospital, CHU Bordeaux, Bordeaux, France; 8 University Paris Cité, INSERM, Inflammation Research Center, Paris, France; 9 Pneumology, Allergology and Transplant Department, AP-HP, Bichat Hospital, Paris, France; 10 Takeda France SAS, Paris, France; 11 Clinsearch, Malakoff, France; 12 National Reference Center for Cytomegaloviruses, Microbiology Department, CHU Limoges, FHU SUPORT, UMR Inserm 1092, Omega Health Institute, Faculty of Medicine-Limoges University, Limoges, France

**Keywords:** antiviral agents, cytomegalovirus, drug resistance, maribavir, transplantation

## Abstract

Maribavir is indicated for the treatment of refractory cytomegalovirus (CMV) infection/disease in patients who have undergone a solid organ transplant (SOT) or hematopoietic cell transplant (HCT). Only limited data on its use in real-world settings have been published from retrospective series. This retrospective study describes the real-world effectiveness of maribavir in 79 transplant patients with refractory CMV infection (67 SOT and 12 HCT) treated under a compassionate use program in France between October 2021 and April 2023. Maribavir was administered for <8 weeks, 8 weeks, and >8 weeks in 17, 32, and 30 patients, respectively. The response rate, defined as viremia clearance, was 53.2%, with a median time to first CMV clearance of 59 days. CMV clearance was observed in patients beyond 8 weeks of treatment. *De novo* maribavir resistance mutations were observed in 13.9% of patients, and CMV recurrence occurred in 45.2% of patients. Presence of CMV disease at baseline was associated with a lower likelihood of maribavir response. Compared to the pivotal SOLSTICE trial, real-world maribavir use demonstrated comparable effectiveness and a lower emergence of maribavir resistance. Moreover, outcomes of patients with a longer treatment duration suggested potential benefits of extending maribavir therapy beyond the recommended 8 weeks.

## Graphical Abstract

**Figure:**
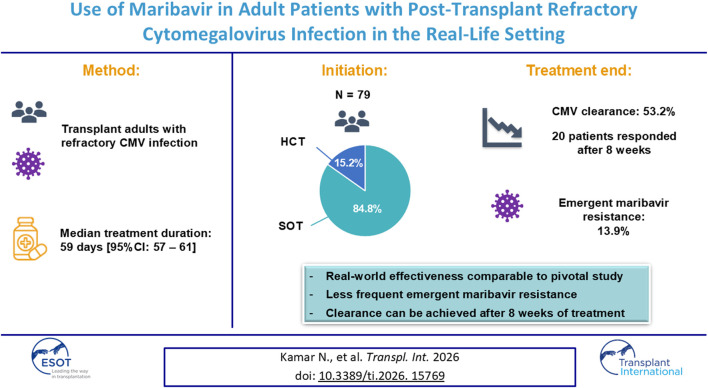

## Introduction

Cytomegalovirus (CMV) is the leading cause of opportunistic infection in recipients of solid organ transplants (SOT) and allogeneic hematopoietic cell transplants (HCT) [1], with CMV infections occurring in 10%–40% of SOT recipients [2] and in approximately half of seropositive allogeneic HCT recipients in the absence of prophylaxis [3, 4].

In 3%–10% of SOT recipients and 11%–50% of HCT recipients, CMV infection is refractory to antivirals, defined as a decrease in blood or plasma viral load of less than one log10 IU/mL after 14 days of antiviral treatment [5, 6]. Refractory CMV can result from host, treatment, or viral factors, including mutations in viral genes that confer drug resistance and possible cross-resistance to antivirals. The most commonly identified drug resistance mutations are located in the genes encoding CMV DNA polymerase (UL54) and CMV protein kinase (UL97) [7]. Refractory CMV infections are associated with increased morbidity and mortality due to the direct and indirect effects of CMV infection, the immunosuppressed status of patients, and the side effects resulting from prior treatment with conventional antivirals [8, 9]. New therapies against CMV are therefore needed to provide alternative approaches to CMV management with the potential to improve patient outcomes [5, 10–12].

Maribavir is an orally bioavailable benzimidazole riboside with potent and selective multimodal anti-CMV activity through competitive inhibition of the CMV protein kinase [13]. The SOLSTICE trial (NCT02931539), a pivotal phase III clinical trial, was designed to evaluate the efficacy and safety of maribavir in adult patients with refractory or resistant CMV infection or disease following transplantation. Patients received maribavir at a dose of 400 mg twice daily for up to 8 weeks. The primary endpoint, CMV viremia clearance at Week 8, demonstrated superior efficacy and lower toxicity of maribavir compared with standard antiviral treatments (ganciclovir, valganciclovir, foscarnet, or cidofovir) for refractory CMV infection or disease with or without antiviral resistance [8, 14].

Maribavir received European marketing authorization on November 11, 2022, for the treatment of CMV infection and/or disease (with or without resistance) that is refractory to one or more prior therapies, including ganciclovir, valganciclovir, cidofovir, or foscarnet, in adult patients who have undergone HCT or SOT. The updated European Conference on Infections in Leukaemia (ECIL) guidelines for the management of CMV in patients after allogeneic HCT strongly recommend the use of maribavir for resistant or refractory CMV infection and disease, highlighting its lower risk of side effects compared to other available treatments [15]. Hence, maribavir can be considered for first-line preemptive therapy in patients with neutropenia or impaired renal function. Furthermore, the Fourth International Consensus Guidelines on the management of CMV in SOT strongly recommend the use of maribavir in patients who are intolerant to valganciclovir or ganciclovir during the treatment phase, as second-line therapy and as the principal alternative in cases of resistance to either ganciclovir or foscarnet [5]. Nevertheless, foscarnet may be preferred over maribavir in clinically severe patients with high viral load.

In France, the compassionate use program (CUP) allowed transplanted patients with refractory CMV infection with or without antiviral resistance to access maribavir (Agence Nationale de Sécurité du Médicament et des produits de santé, ANSM; 2021), prior to European marketing authorization. In accordance with French Health Authority requirements, data on the conditions of prescription and use of maribavir were collected and analyzed within the framework of the CUP. The purpose of the current study is to describe the use of maribavir treatment in a real-world setting.

## Patients and Methods

### Study Design and Setting

This was a retrospective longitudinal study based on the maribavir CUP database. This database was compiled using prospective data collection (from 13 October 2021 to 31 August 2023), in accordance with the therapeutic use protocol (Protocole d’Utilisation Thérapeutique - Recueil des données; PUT-RD) validated by the French Health Authority (ANSM). All French centers had access to the CUP. Requests were submitted by physicians from transplant units and evaluated on a case-by-case basis by ANSM according to the PUT-RD. After inclusion in the CUP, no invasive or specific examinations were performed. All patients were managed by their transplant physicians in accordance with local procedures.

### Ethics

According to French legislation, this study protocol, based on the retrospective analysis of CUP data, was declared to the French authorities under reference methodology MR-004. Patients provided informed consent regarding the use of their data as part of the CUP, and for the reuse of these data for the purposes of this research.

Processing of personal data was performed in accordance with Regulation (EU) No 2016/679 on the General Data Protection Regulation (GDPR) of natural persons.

### Study Population

The patients included in this study were participating in the CUP and completed both treatment initiation and end-of-treatment forms. According to the PUT-RD, the main eligibility criteria to be included in the CUP were: age >18 years, history of a HCT or SOT, ongoing CMV refractory infection (i.e., failed to reach 1 log10 reduction in blood or plasma viral load after 14 days of standard antiviral treatment) with or without antiviral resistance, and estimated glomerular filtration rate (eGFR) >15 mL/min/1.73 m^2^ (calculated using the Modification of Diet in Renal Disease Equation). The definition of refractory infection used in the present study is consistent with recent recommendations [5, 6, 15]. The complete list of inclusion criteria in CUP program is provided in the Supplementary Material.

### Treatment Administration

Maribavir was administered orally at the recommended dose of 400 mg (two 200-mg tablets) twice daily (i.e., total daily dose of 800 mg). Although the recommended duration according to the maribavir label is 8 weeks, the treatment duration could be adjusted by the physician based on the clinical characteristics of each patient.

### Data Collection Timepoints

Data on baseline variables were collected at the time of CUP request or at treatment initiation, then on follow-up visits: at week 4 (W4), W8, W12, and W20. Data on the use of maribavir were collected at each follow-up visit. Given the real-life setting of the study, there were no mandatory visits or interventions. In the event of inconsistencies regarding clinical values, quality controls were conducted by contacting the centers.

### Variables of Interest

Baseline patient characteristics were collected on demographics (age, sex), biological parameters (hemoglobin, leukocyte, and platelet counts, eGFR), transplantation parameters (type and timing), and CMV-related variables (viral load, CMV drug resistance mutations, presence of CMV disease), when available. The collection of all CMV-related variables was not mandatory in routine clinical care.

Viral load levels were measured in local laboratories using a highly sensitive polymerase chain reaction (PCR) with a limit of detection of 200–500 IU/mL to quantify CMV deoxyribonucleic acid (DNA) in blood or plasma, i.e., DNAemia. DNAemia categories were defined as follows:

- High: ≥91,000 IU/mL (4.95 log10) for plasma, or ≥273,000 IU/mL (5.4 log10) for whole blood;
- Intermediate: <91,000 IU/mL (4.95 log10) and ≥9,100 IU/mL (3.95 log10) for plasma, <273,000 IU/mL (5.44 log10) and ≥27,300 IU/mL (4.44 log10) for whole blood;
- Low: <9,100 IU/mL (3.95 log10) and ≥910 IU/mL (2.95 log10) for plasma, <27,300 IU/mL (4.44 log10) and ≥2,730 IU/mL (3.44 log10) for whole blood.

These thresholds were those used in the pivotal SOLSTICE study [14].

No standardized definition of CMV disease was provided in the CUP protocol (PUT-RD); physicians were expected to rely on international recommendations and clinical judgment [16].

Co-prescriptions were recorded at treatment initiation and during follow-up. Treatment duration was measured from therapy start to discontinuation.

For effectiveness assessment, patients were considered treatment responders if they met either of the following criteria: they achieved viremia clearance, defined as a viral load concentration below the lower limit of quantification of <137 IU/mL of plasma, or <411 IU/mL of whole blood); or 2) the viral load concentration was reported as below a threshold determined by the local laboratory or “not detectable” or “not quantifiable”. The proportion of responders was estimated both across the full maribavir treatment period (i.e., between treatment initiation and treatment termination), regardless of treatment duration; and by treatment duration according to three categories: <8 weeks, 8 weeks ±6 days and ≥8 weeks.

Patients were followed for viral load up to 20 weeks after maribavir initiation, which allowed the identification of CMV recurrence after an initial response, defined as a PCR value above reference values for plasma or whole blood. Known CMV drug resistance mutations [17] were identified by Sanger sequencing genotyping before, during, and after maribavir treatment, at the physician’s request.

### Statistical Analysis

Descriptive statistics on patient characteristics, maribavir treatment use, and effectiveness were conducted within the overall study population. Kaplan Meier curves were provided for descriptive purposes. The median time to first clearance and time to treatment discontinuation were calculated, along with their 95% confidence intervals (CI).

Regression models were performed to identify potential predictive factors of treatment effectiveness. The construction of this model included the following steps: 1) selection of clinically relevant variables, 2) univariate analyses, 3) initial multivariate model and 4) final multivariate model. First, clinically relevant variables at treatment request or initiation were considered in univariate analyses [i.e., presence of CMV disease, CMV drug resistance mutations, biological measures (categorized according to population distribution, > or ≤ median), and viral load levels (categorized according to low, intermediate, and high levels)]. Then, univariate analyses were conducted using Chi2 tests or Fisher’s exact test, with variables retained being those with p < 0.05. Statistically significant factors identified in the univariate analyses were then modeled using a Cox regression model to estimate the hazard ratio (HR) adjusted for significantly associated factors (after having verified that they were not highly correlated, i.e., R < 0.7; in case of highly correlated variables, the most significant variables in the univariate analysis were retained in the model). At the end, a final model was obtained considering model performance based on the Akaike information criterion, given the limited sample size.

## Results

### Patient Characteristics

Between October 2021 and April 2023 (18 months), 79 patients initiated treatment with maribavir across 61 transplant departments and completed both initiation and end-of-treatment forms. The completeness rate of collected data exceeded 90% (CUP report ANSM, 2024). At baseline, patients had a mean age of 56.2 years and were predominantly male (69.6%). The majority had received SOT (67 patients; 84.8%), mainly kidney (52; 77.6%), followed by heart (7; 10.4%), lung (7; 10.4%), and pancreas (1; 1.5%). HCT recipients (12 patients) accounted for 15.2% of the study population. Additionally, 18.9% (15 patients) had cytopenia (neutrophil <1,000/mm^3^, platelet <25,000/mm^3^ or hemoglobin <8 g/dL), 16.5% (13 patients) had severe renal impairment (eGFR ≤30 mL/min/1.73 m^2^), and 48.1% (38 patients) had viral loads (DNAemia) in the intermediate to high range (Table 1). Of the 79 infected patients, 57 (72.2%) were asymptomatic, and 22 (27.9%) had CMV disease. Co-prescription of maribavir with other anti-CMV agents was observed in 12 patients, including anti-CMV immunoglobulins Cytotect^®^ (9 patients), foscarnet (2 patients), and cidofovir (1 patient).

**TABLE 1:** Patient characteristics at treatment access request.

Demographics	Patients (N = 79)
Age, years mean ± SD	56.2 ± 13.6
Male/Female sex, n (%)	55 (69.6)/24 (30.4)
Biological parameters	
Absolute neutrophil count,/mm^3^	
<1,000, n/N (%)	8/76 (10.5)
≥1,000, n/N (%)	68/76 (89.5)
Missing data, n	3
Platelet count,/mm^3^	
<25,000, n/N (%)	2/78 (2.6)
≥25,000, n/N (%)	76/78 (97.4)
Missing data, n	1
Hemoglobin level, g/L	
<80, n/N (%)	5/78 (6.4)
≥80, n/N (%)	73/78 (93.6)
Missing data, n	1
Glomerular filtration rate mL/min/1.73 m^2^	
<15, n/N (%)	1/78 (1.3)
15–30, n/N (%)	12/78 (15.4)
>30, n/N (%)	65/78 (83.3)
Missing data, n	1
Solid organ transplant, n (%)	67 (84.8)
Organ transplanted, n/N (%)	
Kidney	52/67 (77.6)
Heart	7/67 (10.4)
Lung	7/67 (10.4)
Pancreas	1/67 (1.5)
Liver	0/67 (0.0)
History of graft rejection, n/N (%)	
No	59/67 (88.1)
Yes	8/67 (11.9)
Hematopoietic stem cell transplant, n (%)	12 (15.2)
Type of hematopoietic transplant, n/N (%)	
Autologous	2/12 (16.7)
Allogeneic	10/12 (83.3)
Graft-versus-host disease (GvHD), n (%)	13 (16.5)
Acute grade > 2, n/N (%)	6/9 (66.7)
Chronic, n/N (%)	3/9 (33.3)
Missing data, n	4
Patients asymptomatic/with CMV disease, n (%)	57 (72.2)/22 (27.9)
Organ infected in patients with CMV disease, n/N (%)	
Gastrointestinal tract (GI) alone or in combination with other symptoms	18/21 (85.7)
Kidney	1/21 (4.8)
Lung	1/21 (4.8)
Eye	1/21 (4.8)
Bone marrow + kidney	1/21 (4.8)
Missing data, n	1
Refractory CMV infections, n	79
Refractory to ganciclovir/valganciclovir, n/N (%)	
Yes	75/78 (96.2)
No	3/78 (3.8)
Missing data, n	1
Refractory to foscarnet or cidofovir, n/N (%)	
Yes	24/74 (34.5)
No	50/74 (65.5)
Missing data, n	5
Analysis of baseline CMV drug resistance mutation, n	79
CMV gene with mutation, n/N (%)	57/79 (72.2)
UL97	51/57 (89.5)
UL54	14/57 (24.6)
Other	7/57 (12.3)
Patients with more than one drug mutation	15/57 (26.3)
Patient with maribavir resistance	1/57 (1.8)
Baseline DNAemia level categories^a^, n	79
High, n/N (%)	18/79 (22.8)
Intermediate, n/N (%)	20/79 (25.3)
Low, n/N (%)	41/79 (51.9)

Abbreviations: CMV, cytomegalovirus; DNAemia; level of human CMV DNA in plasma or whole blood samples; SD, standard deviation.

^a^ The definition of CMV DNAemia level categories is provided in the methods of the article.

Among the 79 patients with refractory CMV, 57 (72.2%) had at least one known CMV drug resistance mutation at baseline (resistant CMV), whereas 22 (27.9%) had no drug resistance mutations detected. One patient presented a mutation conferring resistance to maribavir, while the remaining mutations were associated with resistance to standard antiviral agents. The majority of detected CMV drug resistance mutations were in the UL97 gene, with additional mutations in the UL54 gene, conferring multidrug resistance (Table 1).

### Maribavir Conditions of Use

The mean treatment duration of maribavir was 65.2 ± 4.4 days (median 59 days, [95% CI: 57–61] days). Most patients had a complete 8-week treatment (40.5%, 32 patients) or a treatment prolongation ≥8 weeks (38.0%, 30 patients), while 21.5% of patients (17 patients) received treatment for less than 8 weeks.

### Treatment Effectiveness

**FIGURE 1:**
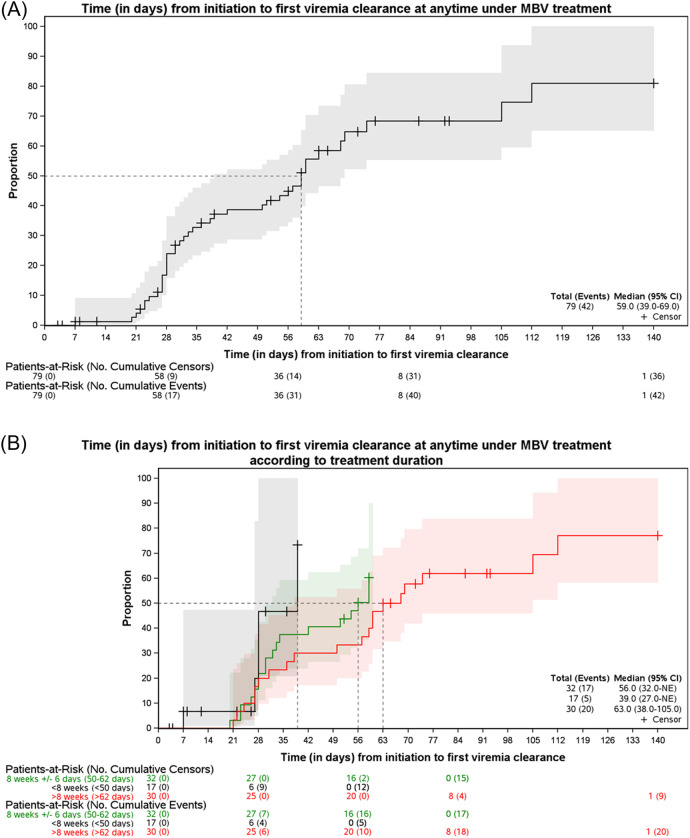
Kaplan Meier estimates of time to first viremia clearance. **(A)** At any time under maribavir (MBV) treatment. **(B)** At any time under maribavir (MBV) treatment, stratified by treatment duration.

Overall, 53.2% of patients (42/79) responded to maribavir at any time during treatment. The median time to first CMV clearance was 59 days [95% CI: 39–69] days (Figure 1A). Moreover, as shown in Figure 1B and Supplementary Table S1, the median time to first CMV clearance according to treatment duration (<8 weeks, 8 weeks ± 6 days, or >8 weeks) was 39, 56, and 63 days, respectively. These data indicate that patients may continue to achieve initial CMV clearance beyond the 8-week mark. Notably, 66.7% (20/30) of patients treated for >8 weeks experienced their first clearance after the 8th week.

Regarding potential predictive factors of treatment effectiveness, clinically relevant variables that could be related to viremia clearance under treatment were first considered in univariate analyses. These analyses showed that only three variables had p-values <0.05: the presence of CMV disease, leucocyte levels, and viral load levels at baseline (Table 2). These variables were then considered in multivariate models. In the final model, the presence of CMV disease at baseline was statistically significantly associated with viremia clearance (Table 3), indicating that patients with CMV disease at baseline were less likely to achieve CMV viremia clearance under maribavir treatment (adjusted HR = 0.44 [95% CI: 0.20–0.96]). However, baseline leucocyte levels (>4,180 mm^3^ vs. ≤4,180 mm^3^) and viral load levels (low vs. intermediate/high) were not statistically associated with response to maribavir: adjusted HR = 1.76 [95% CI: 0.92–3.37] and adjusted HR = 1.56 [95% CI: 0.82–2.97], respectively.

**TABLE 2:** Univariate analysis of factors potentially associated with viremia clearance at any time under maribavir treatment.

Variables	Viremia clearance at any time		Total (N = 79)	P-value
	No (N = 37)	Yes (N = 42)		
Age (year)	​	​	​	0.3211^a^
N (missing)	37 (0)	42 (0)	79 (0)	​
Mean (SD)	54.4 (14.67)	57.7 (12.53)	56.2 (13.59)	​
Range	23.9–78.4	29.3–78.8	23.9–78.8	​
Median (IQR)	57.0 (43.8–65.8)	61.2 (50.1–66.1)	58.6 (47.5–66.1)	​
Gender, N (%)	​	​	​	0.0653^b^
Male	22 (59.5%)	33 (78.6%)	55 (69.6%)	​
Female	15 (40.5%)	9 (21.4%)	24 (30.4%)	​
Type of transplant, N (%)	​	​	​	0.8115^b^
SOT	31 (83.8%)	36 (85.7%)	67 (84.8%)	​
HCT	6 (16.2%)	6 (14.3%)	12 (15.2%)	​
Time between the most recent transplantation and treatment initiation (days)	​	​	​	0.9765^a^
N (missing)	37 (0)	42 (0)	79 (0)	​
Mean (SD)	734.0 (1,303.29)	551.5 (1,007.45)	637.0 (1,151.46)	​
Range	37.0–5,582.0	42.0–6,066.0	37.0–6,066.0	​
Median (IQR)	288.0 (198.0–516.0)	270.0 (201.0–437.0)	270.0 (198.0–485.0)	​
Resistance at initiation of maribavir (CMV drug mutation identified), N (%)	​	​	​	0.3936^b^
No	12 (32.4%)	10 (23.8%)	22 (27.8%)	​
Yes	25 (67.6%)	32 (76.2%)	57 (72.2%)	​
Platelet counts at baseline, N (%)	​	​	​	0.1408^b^
≤157000 mm^3^	22 (59.5%)	18 (42.9%)	40 (50.6%)	​
>157000 mm^3^	15 (40.5%)	24 (57.1%)	39 (49.4%)	​
Leucocyte counts at baseline, N (%)	​	​	​	0.0047^b^
≤4,180 mm^3^	25 (67.6%)	15 (35.7%)	40 (50.6%)	​
>4,180 mm^3^	12 (32.4%)	27 (64.3%)	39 (49.4%)	​
Neutrophil polynuclear at baseline, N (%)	​	​	​	0.2487^b^
≤2,500 mm^3^	21 (58.3%)	19 (45.2%)	40 (51.3%)	​
>2,500 mm^3^	15 (41.7%)	23 (54.8%)	38 (48.7%)	​
Missing	1	0	1	​
ALAT at baseline, N (%)	​	​	​	0.6496^b^
≤25.5 IU/L	17 (47.2%)	22 (52.4%)	39 (50.0%)	​
>25.5 IU/L	19 (52.8%)	20 (47.6%)	39 (50.0%)	​
Missing	1	0	1	​
ASAT at baseline, N (%)	​	​	​	0.9272^b^
≤29 IU/L	19 (51.4%)	22 (52.4%)	41 (51.9%)	​
>29 IU/L	18 (48.6%)	20 (47.6%)	38 (48.1%)	​
Total bilirubin at baseline, N (%)	​	​	​	0.3831^b^
≤7 μmol/L	22 (61.1%)	21 (51.2%)	43 (55.8%)	​
>7 μmol/L	14 (38.9%)	20 (48.8%)	34 (44.2%)	​
Missing	1	1	2	​
Creatinine clearance at baseline, N(%)	​	​	​	0.2163^b^
>90 mL/min	4 (10.8%)	5 (11.9%)	9 (11.4%)	​
60–90 mL/min	11 (29.7%)	10 (23.8%)	21 (26.6%)	​
30–60 mL/min	11 (29.7%)	21 (50.0%)	32 (40.5%)	​
≤30 mL/min	11 (29.7%)	6 (14.3%)	17 (21.5%)	​
Concomitant treatment of CMV, N (%)	​	​	​	0.2646^b^
No	29 (78.4%)	36 (87.8%)	65 (83.3%)	​
Yes	8 (21.6%)	5 (12.2%)	13 (16.7%)	​
Missing	0	1	1	​
Baseline viral load level (High)^c^, N (%)	​	​	​	0.0087^b^
No	26 (70.3%)	39 (92.9%)	65 (82.3%)	​
Yes	11 (29.7%)	3 (7.1%)	14 (17.7%)	​
Baseline viral load level (Intermediate)^d^, N (%)	​	​	​	0.7589^b^
No	25 (67.6%)	27 (64.3%)	52 (65.8%)	​
Yes	12 (32.4%)	15 (35.7%)	27 (34.2%)	​
Baseline viral load level (Low)^e^, N (%)	​	​	​	0.0866^b^
No	23 (62.2%)	18 (42.9%)	41 (51.9%)	​
Yes	14 (37.8%)	24 (57.1%)	38 (48.1%)	​
Baseline viral load levels, N (%)	​	​	​	0.0267^b^
Low	14 (37.8%)	24 (57.1%)	38 (48.1%)	​
Intermediate	12 (32.4%)	15 (35.7%)	27 (34.2%)	​
High	11 (29.7%)	3 (7.1%)	14 (17.7%)	​
Baseline CMV disease, N (%)	​	​	​	0.0230^b^
No	20 (57.1%)	34 (81.0%)	54 (70.1%)	​
Yes	15 (42.9%)	8 (19.0%)	23 (29.9%)	​
Missing	2	0	2	​

Abbreviations: ALAT, alanine aminotransferase; ASAT, aspartate aminotransferase; CI, confidence interval; CMV, cytomegalovirus; HCT, hematopoietic cell transplant; IQR, interquartile range; IU, international unit; SD, standard deviation; SOT, solid organ transplant.

^a^ Kruskal-Wallis p-value.

^b^ Chi-Square p-value.

^c^ The variable “viral load level high” includes the following categories: “Yes” for patients with high viral load levels and “No” for patients with intermediate and low viral load levels.

^d^ The variable “viral load level intermediate” includes the following categories: “Yes” for patients with intermediate viral load levels and “No” for patients with low and high viral load levels.

^e^ The variable “viral load level low” includes the following categories: “Yes” for patients with low viral load levels and “No” for patients with intermediate and high viral load levels.

**TABLE 3:** Results of the multivariate model.

Baseline variables	Hazard ratio (95% CI)
Presence of CMV disease	
No	Reference
Yes	0.44 (0.20–0.96)
Leucocyte levels	
≤4,180 mm^3^	Reference
>4,180 mm^3^	1.76 (0.92–3.37)
Low viral load level	
No (intermediate/high)	Reference
Yes	1.56 (0.82–2.97)

Abbreviations: CI, confidence intervals; CMV, cytomegalovirus.

### Post-Maribavir CMV Mutations and CMV Recurrence

Post-treatment, emergent maribavir resistance was detected in 13.9% (11/79) of patients. All CMV maribavir resistance mutations were located in the UL97 gene and included: T409M (5 patients), F342Y (3 patients), C480F (2 patients), C480R (1 patient), H411L (1 patient), and H411Y (1 patient). Of note, a single patient could present with multiple mutations. The median time to first maribavir resistance mutation occurrence was 45 days. Maribavir resistance mutations occurred during treatment in 8 patients and post-treatment in 3 patients. Among the 42 patients who achieved a treatment response, 19 (45.2%) experienced CMV recurrence within the 20-week follow-up period. The median time to recurrence was 91 days.

## Discussion

Since its marketing authorization in the European Union, maribavir has significantly changed the management of CMV infection and disease due to its efficacy, tolerability, and oral administration. These advantages are reflected in recent updates of international guidelines [5, 15]. Since the pivotal SOLSTICE trial, which demonstrated superior efficacy of maribavir over standard antiviral therapies [14], only a few publications have reported data on maribavir in transplant recipients in real-life settings [18–21].

The national-level retrospective study presented in this article is based on prospectively collected CUP data on the use of maribavir treatment in SOT and HCT recipients with refractory CMV infection or disease. Compared with the SOLSTICE study, the French CUP included patients with more severe hematological and renal impairment. At baseline, several patients in the CUP population presented cytopenia and/or renal impairment, criteria that led to exclusion in the SOLSTICE study. Specifically, 16.5% of patients had severe renal failure and 18.9% had cytopenia, compared with none in the SOLSTICE study. Additionally, more patients in the CUP had high CMV viral load levels (23.0% versus 6.0% in SOLSTICE), and presented with baseline CMV mutations conferring resistance to standard antivirals (72.2% versus 51.5% in SOLSTICE).

The response rate at any time during maribavir treatment in the CUP study was 53.2%, with a median time to first response of 59 days. These findings are consistent with other real-world studies, which reported response rates ranging from 40% to 69% [18–21]. However, the time to response observed in this study was longer than the 22 days reported in the SOLSTICE trial. This difference may be attributed to the absence of standardized PCR testing schedules in the CUP cohort—unlike in the clinical trial—as well as to higher baseline viral loads in CUP patients.

Regarding effectiveness and treatment duration, this real-life study showed that among the 30 patients who received treatment for more than 8 weeks, 67% experienced their first viremia clearance after the 8^th^ week, suggesting that late treatment response can occur beyond the 8-week mark. Furthermore, this analysis indicated that maribavir-emerging resistance during/after treatment occurred in around 14% of patients, which was lower than the 26% reported in the SOLSTICE trial. Other real-world studies have reported varying rates of treatment-emergent resistance, ranging from 8% to 47%, based on small series from single centers [18–20, 22]. Recently, a sub-analysis of the SOLSTICE trial showed that among SOT patients who developed maribavir-resistance, 58% achieved a viral clearance after switching to an alternative therapy [23].

In addition, this analysis describes CMV recurrences regardless of maribavir treatment duration. A potential hypothesis to explain the high percentage of recurrence observed in this study (i.e., 45.2%) could be early treatment discontinuation, as 21.5% of patients stopped treatment before completing 8 weeks. However, these findings cannot be directly compared to those from the SOLSTICE trial since 1) recurrence in SOLSTICE was identified after a fixed treatment duration of 8 weeks; and 2) only clinically significant recurrences (those requiring treatment) were included.

Regarding potential predictive factors of treatment effectiveness, only the presence of CMV disease at baseline emerged as a significant predictor of a lower treatment response. Interestingly, leucocyte or CMV viral load levels at maribavir initiation were not significantly associated with CMV clearance in the final multivariate model. However, given the limited data available in this study to perform predictive modeling, we cannot rule out a potential clinical effect of these variables on CMV clearance following maribavir treatment. Indeed, Kasriel et al. have recently reported that high viral load was significantly associated with a higher rate of recurrence after maribavir treatment and a higher risk of mutation strain emergence [21].

The main study strengths were the study population, the quality of the prospectively collected clinical data, and the analytical approach. Patients were enrolled across the whole country, providing nationwide representativeness of maribavir use in both SOT and HCT populations. In addition, the data collected through the CUP were of high quality, with completeness rates exceeding 90%.

This study has several limitations. Unlike the SOLSTICE pivotal trial [14], in which CMV DNAemia levels were measured in plasma by a centralized laboratory, the CUP cohort data relied on measurements from plasma or whole blood samples tested at individual hospitals. This may have introduced measurement variability. Not all data were available at all timepoints as visits and interventions were not mandatory. Additionally, the follow-up period was limited to 20 weeks after treatment initiation, which precluded long-term assessment of CMV recurrence and maribavir-associated resistance. Finally, the study population included both SOT and HCT patients, with the SOT cohort primarily composed of kidney transplant recipients, which limits the extrapolation of findings to recipients of other organ types.

## Conclusion

Overall, this study provides insight into the real-life use of maribavir in a population with a more severe health profile than in the pivotal SOLSTICE trial [14]. This included more patients with cytopenia, severe renal insufficiency, CMV disease, and elevated viral loads at baseline.

Our findings confirm that maribavir is an effective treatment for transplant recipients with refractory—eventually resistant—CMV infection or disease. The response rate observed was consistent with outcomes from the SOLSTICE trial. However, maribavir resistance occurred less frequently in this cohort, despite patients being more severely ill at baseline. Moreover, this study highlights the need for adaptable treatment duration, with potential benefits in extending maribavir therapy beyond the recommended 8 weeks to accommodate late treatment responders. Further studies are still needed to assess long-term outcomes in transplant recipients receiving anti-CMV therapy with maribavir.

## Data Availability

The original contributions presented in the study are included in the article/Supplementary Material, further inquiries can be directed to the corresponding author.
