# Supplementary material for: Use of Maribavir in Adult Patients With Post-Transplant Refractory Cytomegalovirus Infection in the Real-Life Setting
Source: Transpl Int. 2026 Feb 19;39:15769. doi: 10.3389/ti.2026.15769 (PMC12960281; doi:10.3389/ti.2026.15769)
Supplement: Supplementary file 1 [file Supplementaryfile1.docx]

# Supplementary Material

# Capsule Sentence Summary

The real-world effectiveness of maribavir treatment in adults with post-transplant refractory cytomegalovirus infection was comparable to that of the pivotal study. Extending the treatment duration beyond the recommended 8 weeks may provide further benefits in curing the disease.

# Methods

Eligibility criteria of patients with refractory cytomegalovirus (CMV) infection/disease in the compassionate use program (CUP):

The patient must imperatively meet all of the following criteria:

• age ≥ 18 years

• weight ≥ 50 kg

• The patient has a hematopoietic stem cell transplant or solid organ(s) transplant

• The patient has a documented CMV infection on two successive samples taken at least 1 day apart, with a viral load, determined by quantitative PCR or comparable quantitative CMV DNA results, corresponding to a value:

≥ 2730 IU/mL for whole blood level

or

≥ 910 IU/mL for plasma level

• The patient has an ongoing refractory CMV infection, defined as the inability to achieve a decrease in viral load of at least 1 log10 (common logarithm base 10) of whole blood or plasma CMV DNA level after a treatment period of 14 days or more, with at least one of the following treatments: IV ganciclovir / oral valganciclovir, IV foscarnet or IV cidofovir.

• The patient presents the following results in biological assessments:

- Neutrophils ≥1000/mm^3^ [1.0 x 10^9^/L]

- Platelets ≥25000/mm^3^ [25 x 10^9^/L]

- Hemoglobin ≥8 g/dL

- Estimated Glomerular Filtration Rate (eGFR) >30 mL/min/1.73m² calculated according to the Modification of Diet in Renal Disease (MDRD) formula.

• The patient does not have invasive CMV disease of the central nervous system.

Note: a patient presenting with refractory CMV viremia AND CMV retinitis may be considered if:

- all eligibility criteria are met AND

- the CMV retinitis will be treated with an agent other than maribavir (except ganciclovir/valganciclovir).

• The patient does not have serum transaminases (ASAT and ALAT) >5 times the upper limit of normal (ULN), or total bilirubin ≥3.0 x ULN (except in the case of documented Gilbert's syndrome).

Note: patients whose biopsy has confirmed CMV hepatitis may still be considered despite ASAT or ALAT levels >5 times ULN.

• The patient does not suffer from severe episodes of vomiting, diarrhea or other serious gastrointestinal disease in the 24 hours preceding the first dose of treatment.

• The patient has never been treated with maribavir previously.

• The patient can swallow tablets whole, crushed or administered using an oro-gastric or naso-gastric tube.

• The patient (male/female) commits to abstain from all sexual activity or to use an acceptable method of contraception (for example, intrauterine device, double barrier, hormonal contraception in combination with another acceptable method), as determined by the treating physician, during the period of maribavir administration and for 3 months following the end of treatment.

• The female patient, if of childbearing age, must not be pregnant or breastfeeding.

• The patient is not HIV seropositive (no documented seropositivity).

• The patient is not receiving treatment for acute or chronic hepatitis C.

# Supplementary Tables

**Table S1: Kaplan Meier estimates of time to first viremia clearance at any time under maribavir treatment, stratified by treatment duration**

|  | **Event/**  **Total** | **Median (95% CI)^1^** | **Survival estimates (95% CI)^1^** | **Covariate Level**  **p-values** | **p-value** |
| --- | --- | --- | --- | --- | --- |
| **Duration of treatment (days)** |  |  |  |  | **0.1694^3^** |
| 8 weeks ± 6 days (50-62 days) | 17/32 | 56.0 (32.0-NE) | 0 days: 0.0 (NE-NE%) | 0.2794^4^ |  |
|  |  |  | 28 days: 21.9 (11.2-42.6%) |  |  |
|  |  |  | 56 days: 50.4 (35.3-71.8%) |  |  |
|  |  |  | 84 days: NE (NE-NE%) |  |  |
|  |  |  | 140 days: NE (NE-NE%) |  |  |
| <8 weeks (< 50 days) | 5/17 | 39.0 (27.0-NE) | 0 days: 0.0 (NE-NE%) | 0.0454^4^ |  |
|  |  |  | 28 days: 46.7 (20.6-100.0%) |  |  |
|  |  |  | 56 days: NE (NE-NE%) |  |  |
|  |  |  | 84 days: NE (NE-NE%) |  |  |
|  |  |  | 140 days: NE (NE-NE%) |  |  |
| >8 weeks (> 62 days) | 20/30 | 63.0 (38.0-105.0) | 0 days: 0.0 (NE-NE%) | -- |  |
|  |  |  | 28 days: 20.0 (9.6-41.5%) |  |  |
|  |  |  | 56 days: 33.3 (19.9-55.9%) |  |  |
|  |  |  | 84 days: 61.9 (45.8-83.7%) |  |  |
|  |  |  | 140 days: 77.2 (58.2-100.0%) |  |  |

Abbreviations: CI, confidence intervals; NE, not evaluated.

^1^ Cumulative incidence method; ^2^ Cox model; ^3^ Gray’s k-sample test for equality of cumulative incidence functions; ^4^ Wald Chi-Square test
